# Genome-Scale Identification of Membrane-Associated Human mRNAs

**DOI:** 10.1371/journal.pgen.0020011

**Published:** 2006-01-13

**Authors:** Maximilian Diehn, Ramona Bhattacharya, David Botstein, Patrick O Brown

**Affiliations:** 1 Department of Radiation Oncology, Stanford University School of Medicine, Stanford, California, United States of America; 2 Department of Biochemistry, Stanford University School of Medicine, Stanford, California, United States of America; 3 Department of Genetics, Stanford University School of Medicine, Stanford, California, United States of America; 4 Lewis-Sigler Institute for Integrative Genomics, Princeton University, Princeton, New Jersey, United States of America; 5 Howard Hughes Medical Institute, Stanford University School of Medicine, Stanford, California, United States of America; North Carolina State University, United States of America

## Abstract

The subcellular localization of proteins is critical to their biological roles. Moreover, whether a protein is membrane-bound, secreted, or intracellular affects the usefulness of, and the strategies for, using a protein as a diagnostic marker or a target for therapy. We employed a rapid and efficient experimental approach to classify thousands of human gene products as either “membrane-associated/secreted” (MS) or “cytosolic/nuclear” (CN). Using subcellular fractionation methods, we separated mRNAs associated with membranes from those associated with the soluble cytosolic fraction and analyzed these two pools by comparative hybridization to DNA microarrays. Analysis of 11 different human cell lines, representing lymphoid, myeloid, breast, ovarian, hepatic, colon, and prostate tissues, identified more than 5,000 previously uncharacterized MS and more than 6,400 putative CN genes at high confidence levels. The experimentally determined localizations correlated well with in silico predictions of signal peptides and transmembrane domains, but also significantly increased the number of human genes that could be cataloged as encoding either MS or CN proteins. Using gene expression data from a variety of primary human malignancies and normal tissues, we rationally identified hundreds of MS gene products that are significantly overexpressed in tumors compared to normal tissues and thus represent candidates for serum diagnostic tests or monoclonal antibody-based therapies. Finally, we used the catalog of CN gene products to generate sets of candidate markers of organ-specific tissue injury. The large-scale annotation of subcellular localization reported here will serve as a reference database and will aid in the rational design of diagnostic tests and molecular therapies for diverse diseases.

## Synopsis

An important goal of current biological research is annotation of human genes with relevant descriptors and properties. One critical property of interest to biologists and medical researchers is the subcellular localization of gene products, as this affects a protein's biological role and our ability to use it as a therapeutic target. This study used a microarray-based functional genomic method that allows rapid, large-scale identification of subcellular localization, enabling the authors to annotate the localization of thousands of previously uncharacterized human gene products. The authors then provide an example of how these data can be used by applying them to the search for tumor-specific markers. Using data from hundreds of DNA microarray profiles of global gene expression patterns in tumors and normal tissues, they identify candidate genes encoding membrane-associated and secreted proteins that are highly overexpressed in tumors and that might therefore be particularly good targets for diagnostic tests or molecular therapies. Diagnostic tests based on these markers could potentially enable cancers to be detected earlier than is currently possible, and molecular therapies targeting the products of these genes could have high specificity for the corresponding cancers.

## Introduction

The subcellular localization of proteins critically affects their biological roles and functions. For example, sequence-specific DNA-binding proteins can only alter transcriptional activity if they are localized to the nucleus, and transmembrane (TM) receptors can only bind their soluble ligands if they are at least partially exposed to the extracellular environment. Categorization of proteins by subcellular localization is therefore one of the essential goals for functional annotation of the human genome.

Proteins that are inserted into membranes or secreted are a particularly important class, as they include signal transduction receptors, transporters, channels, cell-to-cell signaling molecules, extracellular matrix components, and adhesion molecules. Surface and secreted proteins are also of special relevance to many areas of medicine. Plasma membrane proteins and secreted signaling proteins are candidate targets for monoclonal antibody-based therapies [1]. There are already more than ten Food and Drug Administration-approved monoclonal antibody therapeutics and dozens more in clinical development. Some of these, including Trastuzumab (Herceptin), Rituximab (Rituxan), and Cetuximab (Erbitux), target TM proteins on the surface of malignant cells and have firmly established their value in the treatment of cancer. Secreted and shed proteins with tumor- or disease-specific expression patterns represent potential targets for diagnostic assays in biological fluids. Such assays are currently being used to screen patients for diagnosis or detection of recurrence of a number of malignancies, including prostate, ovarian, and liver cancer [2]. Conversely, intracellular proteins that are released into the extracellular space with cell injury or death provide the basis for sensitive assays to diagnose specific organ injury (e.g., troponins, creatine kinase, myosin, and transaminases) [3]. Large-scale identification of surface, secreted, and intracellular proteins that are specific to organs, tissues, or disease thus has great potential value in facilitating the further development of these therapeutic and diagnostic approaches.

A variety of empirical and computational approaches has been developed for identifying membrane and secreted proteins. Commonly employed experimental approaches include the signal-sequence trap [4,5], the signal-exon trap [6], and construction of cDNA libraries from membrane-associated mRNAs [7]. Computational approaches for predicting the presence of signal peptides (SPs) in protein sequences have used weight matrices, artificial neural networks, and hidden Markov models (reviewed in [8]). Similarly, algorithms to identify putative TM domains include sliding window methods, neural networks, and hidden Markov models (reviewed in [9]). Both the experimental and the computational approaches are imperfect, and combining information from different methods will likely improve sensitivity and specificity. In that respect, a major limitation of most of the empirical methods is that they are not designed to be used on a genome-wide scale. Although the existing methods focus on identifying membrane-associated/secreted (MS) proteins, it would also be useful to systematically identify probable cytosolic/nuclear (CN) proteins.

We set out to classify thousands of human genes as encoding either MS or CN proteins, using a previously described genome-scale method [10]. Briefly, this method takes advantage of the consistent (though not universal [11]) difference in the subcellular location at which these two classes of proteins are translated. Most MS proteins are translated by polyribosomes bound to the cytoplasmic face of the endoplasmic reticulum (ER), while most CN proteins are translated on polyribosomes in the cytoplasm. The mRNAs corresponding to these two classes of proteins can therefore be separated by sedimentation equilibrium based on their association with microsomes [12] and can subsequently be analyzed using cDNA microarrays.

Using this approach, we identified more than 5,000 putative MS and more than 6,400 putative CN genes (UniGene clusters). Our annotations agreed well with in silico methods for predicting localization, but also included thousands of genes for which such predictions were not available. One advantage of our approach was the ability to directly apply our categorizations to microarray gene expression data from hundreds of primary tumor and normal tissue samples that were generated separately using the same DNA microarrays. We were thus able to identify hundreds of genes that showed tumor-specific expression patterns and that are highly likely to encode MS proteins. These data provide a foundation for the development of targeted therapies and diagnostic tests for a wide variety of human malignancies.

## Results

### Identification of MS- and CN-Encoding Genes

Based on previously reported microarray experiments [13], we chose a panel of 11 cell lines whose gene expression patterns would, in aggregate, encompass a large set of mRNAs (Table S1). RNAs in the membrane and cytosolic fractions of each cell line were separated using a previously described sedimentation equilibrium method [10], and fluorescently labeled cDNA was generated from each fraction. For every cell line, we labeled cDNA from membrane-associated RNA with Cy5 and cDNA from cytoplasmic RNA with Cy3, mixed these, and hybridized them to DNA microarrays containing up to approximately 42,000 elements (representing approximately 32,500 UniGene clusters). For a subset of the cell lines, we first amplified the fractionated RNAs using a linear amplification method [14]. Several cell lines were fractionated more than once; the results from a total of 19 hybridizations were used for further analysis.

**Figure 1:**
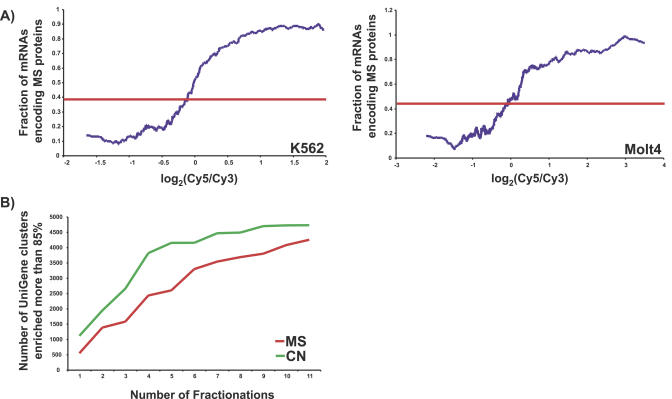
DNA Microarray Analysis of Subcellular mRNA Populations (A) Moving average analyses of the fraction of mRNAs encoding MS proteins. Data for two representative fractionations are shown. In each case, well-measured array elements representing characterized genes were extracted, and the local enrichment for MS-encoding genes (window size = 151) was calculated as a function of the Cy5/Cy3 ratio. The horizontal line represents the overall fraction of MS genes on the microarrays used in these experiments. (B) Discovery rate analysis for the identification of MS and CN genes. A representative microarray hybridization was chosen for each cell line and the total nonredundant number of classified MS or CN genes (UniGene clusters) was calculated after each new fractionation.

The thousands of genes represented on the microarrays for which the subcellular localizations of the protein products have previously been identified provided internal standards for assessing the success of each fractionation and for determining the confidence with which an uncharacterized gene could be classified as MS or CN. Genes encoding proteins inserted into cellular membranes or that are secreted were designated as the “MS reference set,” while genes encoding cytoplasmic or nuclear proteins were designated as the “CN reference set.” Limiting our search to the 24,365 UniGene clusters that were detectably expressed in at least three of the samples, we used empirical localization data reported in the Swiss-Prot [15] and LocusLink [16] databases to identify 2,701 known MS clusters and 2,744 known CN clusters. We then estimated the percentage of mRNAs encoding MS proteins as a function of the Cy5/Cy3 ratio for each array, based on the data for these reference gene sets [10]. Figure 1A displays the results of this analysis for two of the fractionations. Genes for which we found the highest Cy5/Cy3 ratios were highly enriched for those encoding known MS proteins, while genes with the lowest Cy5/Cy3 ratios were highly enriched for those encoding CN proteins.

To estimate the number of novel MS or CN genes that were identified by each additional fractionation, we calculated the total number of unique cDNA clones that were more than 85% enriched in the membrane or cytosolic fraction on at least one array. As show in Figure 1B, the rate at which additional genes were classified dropped off significantly with each successive cell line analyzed. Nevertheless, a considerable number of novel genes continued to be classified with each new analysis, and these genes are likely to encode cell-type-specific markers that were not expressed in the preceding cell lines. It is likely that by extending our approach to include even more cell lines or tissues, we could substantially increase the total number of genes classified as MS or CN.

**Figure 2:**
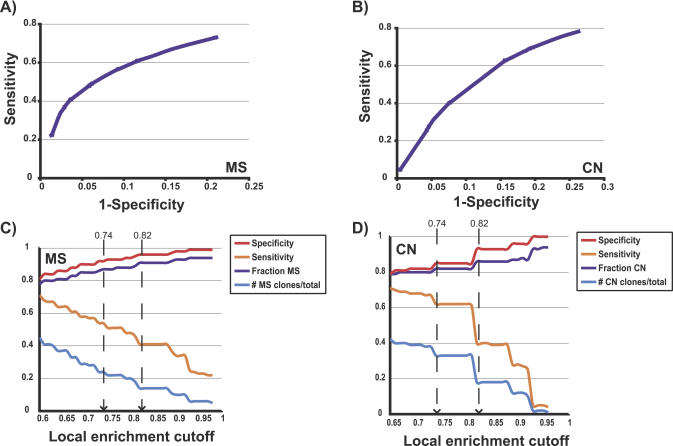
Large-Scale Categorization of MS and CN Genes We evaluated the ability of various array element-specific parameters to classify genes encoding MS (A) or CN (B) proteins, using receiver operator analysis. Based on these analyses, we chose the average log_2_ Cy5/Cy3 ratio for assigning the final localization annotations (see text and Protocol S1). The curves were generated by incrementally relaxing the parameter cutoff values to generate gene sets with varying fractions of known MS- or CN-encoding genes. (C) Relationship between sensitivity, specificity, fraction of characterized genes encoding MS proteins, and the total number of clones classified as MS, using the average log_2_ Cy5/Cy3 ratio as the selection criteria. The vertical arrows indicate two cutoffs used for subsequent analyses. (D) Same as in (C) but for genes encoding CN proteins.

In order to enable more complex analyses, we next attempted to catalog the largest possible number of MS and CN genes. To do so, we calculated various descriptive statistics (median, mean, minimum, maximum, etc.) for a number of parameters for every clone across all arrays, including: the local percentage of characterized MS genes based on the moving average analysis (see above), the base 2 logarithm of the Cy5/Cy3 ratio, the ratio of intensity to local background for both Cy3 and Cy5, and the background-subtracted intensity for both Cy3 and Cy5. As a final parameter, we included the ratio of the sum of Cy5 background-corrected intensities to the sum of Cy3 background-corrected intensities across all arrays. To identify the best classification approach, receiver–operator curves were generated using each of these parameters (Figure 2A and 2B and data not shown). Based on these calculations, we chose the average log_2_ Cy5/Cy3 ratio as the metric for our subsequent analyses. Clones were ranked in descending order of the average log_2_ Cy5/Cy3 ratio, and a moving average approach was used to identify the local percentage of characterized MS/CN proteins at each end of this distribution. Since a subset of the UniGene clusters included on the arrays was represented by two or more elements, we removed all clusters with ambiguous localizations (i.e., clusters that contained clones classified as both MS and CN). As expected, relaxing the local percentage enrichment allowed annotation of an increasingly larger set of unknown clones, but at the expense of specificity (Figure 2C and 2D). Based on these results, we chose two enrichment cutoffs for further analysis (see Dataset S1), and the results for the less stringent of these are summarized in Table 1. Including genes with known localization, we were able to classify 60% of UniGene clusters that passed minimum expression-level filters in at least one of the fractionations (15,360 out of 24,365).

**Table 1:**
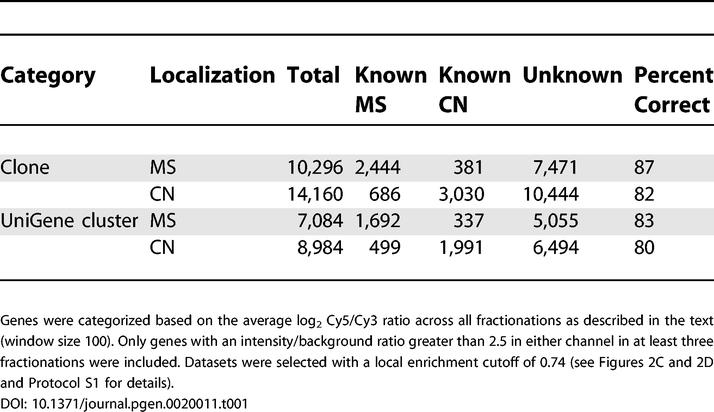
Experimental Annotation of MS and CN Genes

### Comparison of In Silico and Empirical Classifications

**Figure 3:**
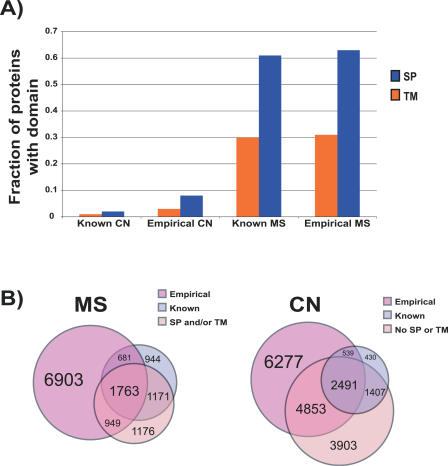
Comparison of Empirical Classifications of MS and CN Genes with In Silico Prediction Methods (A) We were able to retrieve curated, NP protein accessions for 5,504 of the well-measured UniGene clusters on our arrays. The prediction algorithms used were SignalP (HMM/Smean score method) [33] for SPs and TMHMM (first60 score cutoff greater than 10) [34] for TM domains. In order to calculate the fraction of proteins within a category that contained a given motif, the overlap between that category and the genes with protein sequences was used. (B) Venn diagrams showing the overlap between the empirically determined cDNA clones, clones with in silico predictions, and clones encoding proteins with known subcellular localization. For this analysis, we were able to retrieve representative protein accessions for 10,006 cDNA clones from UniGene and applied the prediction algorithms as in (A).

We next compared our classifications with in silico algorithms for protein localization prediction. Algorithms that predict TM domains and SPs are particularly relevant to the distinction between CN and MS proteins. Using all UniGene clusters for which we obtained high-quality data and for which full-length, curated NP protein sequences were available in LocusLink, we analyzed the concordance between our empirical method and the two prediction algorithms. As shown in Figure 3A, there was a strong association between the distribution of predicted SPs and TM domains and our empirical annotations. The putative MS proteins identified by our empirical method contained a slightly higher fraction of predicted TM domains and SPs than the set of MS proteins curated from published sources. For the set of CN proteins identified by our method, the fraction that were predicted to contain TM domains or SPs was marginally higher than the set of CN proteins curated from published sources, consistent with the slightly higher rate of contamination seen in Table 1. This striking concordance between the two approaches corroborates the accuracy of our experimental annotations. As Figure 3B indicates, the empirically determined, in silico-predicted, and curated/published sets were overlapping but not identical, and thousands of clones that could not be classified by the known or in silico methods were assigned with high confidence by our empirical method. Furthermore, it is important to note that even among previously known MS proteins, only 64% contained TM domains and/or SPs, underscoring the limited sensitivity of the computational methods and the need for experimental methods that can efficiently identify this class of proteins.

### Analysis of mRNAs with Unexpected Localization

While the majority of the mRNAs that encode known CN or MS proteins were found in the expected fraction by our procedure, the RNAs encoded by a small number of known genes were assigned to the seemingly incorrect fraction. We used GO-TermFinder [17] to measure the enrichment of Gene Ontology annotations among this group. The subset of genes whose protein products have been reported to be CN and that were classified as MS by our method was not enriched for any GO term, suggesting that, at least by this metric, they did not differ significantly from all other CN genes. We focused further on the known CN genes that were represented by multiple clones on the arrays and for which the majority of clones were classified as MS by our method. On further investigation, we found the curated information on localization of the products of many of these genes to be incomplete or incorrect. For example, stomatin, an integral membrane protein exposed on the cytoplasmic surface of red blood cells, and secretagogin, a secreted protein, carried GO annotations suggesting cytoplasmic localization even though evidence exists in the literature that they are most likely translated on the rough ER [18,19]. This suggested that additional curation to refine and correct annotations of the known gene sets could increase the number of unknown genes that can be annotated by the microarray method.

A second class of CN genes with discrepancies between their reported localization and our results consisted of genes that belonged to families in which other members had documented MS localization. For example, retinol-binding protein 5 (RBP5) was classified by our analysis as MS, but the protein is cataloged as cytoplasmic by Gene Ontology. While no other experimental data suggesting MS localization for RBP5 have been reported, the closely related protein RBP4, the serum retinol carrier, is a documented secreted protein [20]. This suggests that RBP5 may also have a, as yet unrecognized, secreted form.

A third class of CN genes encoding proteins reported to be cytoplasmic or nuclear, but whose mRNAs fractionated with membranes in our analysis, included genes encoding proteins that function at or near cellular membranes, but are not integral membrane components themselves. ACK1 and TXK, two nonmembrane spanning kinases, were examples of this class. The protein encoded by ACK1 is localized to clathrin-containing vesicles [21], and the TXK protein binds lipid rafts via a palmitoylated cysteine-string motif [22]. Other examples included cyclin B3, whose family member cyclin B1 interacts with the cytoplasmic domain of the TM receptor PTCH [23], and cyclin E2, which has been shown to associate with endosomes and the plasma membrane of hepatocytes [24]. Thus, some genes, previously reported to be CN proteins and whose mRNAs were enriched in the membrane fraction, encode proteins that function in the cytosol, but in close association with membranes. The membrane association of their transcripts may therefore reflect a novel RNA sorting process that localizes the polyribosomes carrying mRNAs encoding these proteins to membranes. In support of this idea, recent evidence from yeast indicates that some RNA-binding proteins preferentially associate with 3′ untranslated regions of mRNAs encoding membrane-associated proteins [25]. A further mechanism by which proteins containing membrane-binding domains or modifications (pleckstrin homology domain, myristoylation, etc.) might be translated by membrane-associated polyribosomes could involve the cotranslational maturation of the membrane-binding domain, resulting in recruitment of the polyribosomes to membranes.

Another potential explanation for unexpected enrichment of some CN gene transcripts in the membrane fraction applies to genes whose transcripts are alternatively spliced, with one splice form encoding a CN protein, while another encodes an MS protein. This is the case for estrogen receptor 1 (ESR1), which is generally classified as encoding a CN protein, but which our method classified as MS. While the best characterized role for ESR1 is as a transcriptional activator in the nucleus, several reports provide evidence for an alternatively spliced variant that encodes a surface-exposed membrane-bound protein that can participate in signal transduction [20,26]. Interestingly, we also found the mRNA encoding the progesterone receptor (PGR) to be enriched in the MS fraction, suggesting that there may also be a membrane-bound form of this nuclear hormone receptor.

We also investigated genes whose products were reported to be secreted or localized to membranes but whose transcripts were enriched in the CN fraction. Using GO-TermFinder (Table S2) we found that this group of genes was statistically significantly enriched for being involved in vesicle and protein transport when compared to all MS genes. Even more strikingly than with the anomalously localized CN genes, a large fraction of the apparently mislocalized transcripts were actually due to incorrect assignment of genes to the MS group. These misannotated genes included many genes encoding cytoplasmic proteins that function at or near membranes, such as GOSR2, STX17, and GNAS. Furthermore, genes encoding proteins localized to peroxisomes were highly overrepresented in this class. Both peroxisomal membrane and matrix proteins have been shown to be translated on free polyribosomes in the cytoplasm and are posttranslationally inserted into peroxisomes [27]. Therefore, these proteins never traverse the rough ER, consistent with our observation of peroxisomal mRNAs in the cytosolic fraction. The similar, unexpected enrichment of mRNAs encoding vesicle transport proteins in the CN fractions suggests that a subset of these proteins may also be incorporated into membranes posttranslationally, rather than entering them via the standard ER cotranslational insertion mechanism. Such a posttranslational translocation pathway for ER import has been previously reported and may be present in all eukaryotes [28]. It is interesting to note that the mRNA encoding SEC61B, one of the components of the complex that binds ribosomes and serves as the channel by which proteins are cotranslationally inserted into the ER, was in this group.

Another class of membrane or secreted proteins whose mRNA reproducibly fractionates with the cytosol was represented by the small inducible cytokine subfamily E, member 1 (SCYE1). This gene encodes a multifunctional polypeptide with both cytokine and tRNA-binding activities. Studies have shown that the tRNA-binding domain interacts with aminoacyl-tRNA synthetases, while a different domain of the protein, with inflammatory cytokine activity, is released extracellularly by proteolysis in response to apoptotic stimuli [29]. These examples indicate that a discrepancy between the localization of an mRNA and that of its protein product can point to important aspects of a gene's biological role. Further analysis of this group of mRNAs is therefore likely to uncover new features of the subcellular localization of human mRNAs.

### Estimation of the Fraction of MS Genes in the Human Genome

A particularly striking result of these studies was that 44% of the genes that we could classify were predicted from our results to encode MS proteins (Table 1). Previous estimates of the fraction of human genes that encode membrane or secreted proteins are based on in silico predictions of SPs and TM domains and have ranged from 15% to 30% [30–32], implying that a substantial majority of genes encode proteins localized in the nucleus or cytoplasm. In our own in silico analyses using SignalP [33] and TMHMM [34], we found that approximately 25% to 30% of full-length protein sequences curated in LocusLink contained either a predicted TM domain, an SP, or both. We attempted to estimate this fraction more rigorously using our experimental data and the following equation:


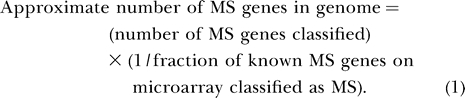


This allowed us to estimate the number of genes encoding MS proteins we would have expected to find had we been able to identify every one of them. In order to assess the robustness of our estimate, we performed the calculation with two MS gene sets, one derived from lower and the other from higher stringency analyses of our localization data. Using both sets, we estimated the total number of MS UniGene clusters present on our arrays to be approximately 7,000. Given that our arrays provided technically adequate data for 24,365 clusters, and assuming that these were representative of all genes in the genome, our data suggest that approximately 30% of human genes encode MS proteins. Thus, our empirical results are consistent with the upper extreme of the previous computational predictions. This analysis implies that there are likely to be thousands of still unrecognized surface-exposed and secreted proteins that could serve as useful diagnostic markers or therapeutic targets.

### Membrane-Associated Tissue- and Tumor-Specific Markers

We were particularly interested in identifying potential secreted or surface-exposed TM proteins that were more highly expressed in one or more cancers than in most or all normal tissues. These proteins are potential diagnostic markers or targets for monoclonal antibody-based therapies. We began with a list of putative membrane or secreted proteins identified in this work (see http://microarray-pubs.stanford.edu/mbp2). We removed from this list all of the known genes encoding CN proteins that we had curated earlier. We next added any gene encoding a membrane or secreted protein that was identified by our previous database searches but was not identified as such in our analysis. This aggregate list contained approximately 7,300 putative genes (UniGene clusters), represented on our microarrays by 12,030 cDNA clones (see Dataset S2).

The same DNA microarrays that we used to catalog genes encoding MS or CN proteins have been used to profile gene expression in hundreds of human tumor samples and normal tissues. We therefore assembled data from 745 microarray analyses of human tumors and normal tissues, including malignancies of the brain, breast, kidney, lung, stomach, ovary, pancreas, soft tissues, testis, and hematopoietic system [35–49], all of which were analyzed by comparative hybridization of tumor or normal mRNA with the same common reference RNA. Using hierarchical clustering [50] and our aggregate MS gene list, we compared expression of genes encoding MS proteins among these samples.

In order to examine the relationship between tumor and normal specimens stemming from the same tissue, we first clustered centroid array vectors for each tumor and normal class. Tumor and normal classes from the same tissue generally clustered together on terminal branches, with a few exceptions (see Figure S1). When individual arrays were clustered, all tumor types formed discrete clusters, and all but a few specimens clustered in these defined tumor groups. Tumor samples clustered near the corresponding normal samples from the same tissue but generally did not intermingle (data not shown). This indicates that the expression patterns of genes encoding membrane or secreted proteins define molecular signatures that can identify the tissue origin of tumors and that the membrane compartments of the tumors tend to be similar to, but distinct from, those of the normal tissues from which they arose.

**Figure 4:**
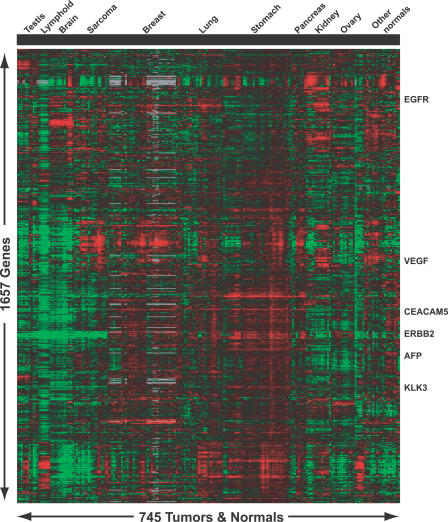
Expression of MS Genes in Human Malignancies and Normal Tissues Gene expression profiles for 745 tumor and normal specimens were generated on the same types of microarrays used for the fractionation experiments. Array elements representing MS genes that varied more than 3-fold from the median on at least three microarrays were included. The data are displayed as a hierarchical cluster where rows represent genes (UniGene clusters) and columns represent experimental samples. Colored pixels capture the magnitude of the response for any gene, where shades of red and green represent induction and repression, respectively, relative to the median for each gene. Black pixels reflect no change from the median and gray pixels represent missing data. For clarity of display, tumor and normal samples for each tumor type were hierarchically clustered separately and then arranged by the order derived from clustering their mean centroids (see Protocol S1). The positions of several genes are indicated.

Figure 4 depicts the diverse, complex patterns of variation in MS gene expression in the human tumor and normal tissues that we examined. A significant fraction of these genes were expressed in tissue-specific patterns, reflecting the vast qualitative differences between the membrane compartments of cells stemming from different tumors. Genes encoding a number of MS proteins that are currently used as cancer markers or targets of therapeutic antibodies were identified by our approach, including *KLK3 (PSA), AFP, EGFR, ERBB2, CEACAM5 (CEA),* and *VEGF.* Their expression patterns were generally consistent with previous studies. For example, *ERBB2* was especially highly expressed in a subset of breast tumors, while *CEACAM5* was most highly expressed in several epithelial tumors, including those of the breast, stomach, and lung [51–53].

### Identification of MS Tumor Markers

We next wished to identify potential therapeutic and diagnostic targets that were expressed in a tumor-specific fashion. Small molecule or monoclonal antibody-based therapies have shown promise as single agents or in conjunction with traditional modalities such as chemotherapy and radiotherapy, and it is likely that even better tumor control and cure rates could be achieved by developing combinations of biologically based drugs for each tumor type. The ideal class of markers for this approach consists of surface-exposed and secreted proteins that are highly expressed in tumor cells and only minimally expressed in normal tissues. The combination of our MS gene list and the large-scale gene expression dataset we constructed allowed us to rationally identify such candidate genes. We ranked genes based on the difference between the median expression in tumor samples of a given class and the 95th percentile expression level across all normal tissue samples. This resulted in selection of genes that were more highly expressed in most of the tumor samples than in the vast majority of normal tissues. To further prioritize candidate genes, we also incorporated an estimate of transcript abundance into our selection scheme, hypothesizing that more highly expressed genes will make better therapeutic or diagnostic targets. Since all of the microarray data presented here were generated using a two-color comparative hybridization approach that produces measurements of relative abundance between different samples, we aimed to identify the relative transcript abundance of potential candidate markers within each tumor class. To estimate transcript abundance, we used data from comparative hybridizations of the common reference RNA used in each tumor experiment versus normal female genomic DNA [48]. These data reflected the relative abundance of each transcript in the reference and were used to calculate a relative transcript abundance index for each gene within each tumor subtype (see http://microarray-pubs.stanford.edu/mbp2). This information can be used to help prioritize genes for follow-up studies.

**Figure 5:**
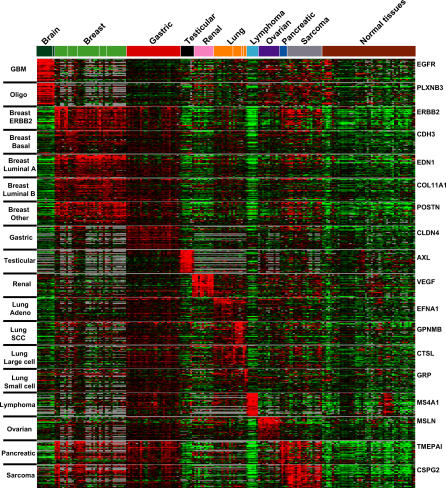
Identification of MS Tumor Markers Array elements were ranked based on the difference between the median expression in tumor samples of a given class and the 95th percentile expression level across all normal tissue samples. The dataset was selected in a similar fashion as for Figure 4 (see Protocol S1). Only array elements that passed data quality filters for at least 40% of normal tissues and at least 50% of one or more tumor classes were considered. The top 50 genes for each tumor class are shown, and the positions of several genes are indicated. Brain, lung, and breast tumors were divided into their previously known histologic and molecular subtypes. GBM, glioblastoma multiforme; oligo, oligoastrocytoma/oligodendroglioma; adeno, adenocarcinoma; SCC, squamous cell carcinoma.

As shown in Figure 5, our approach identified a number of genes that encode proteins that are targets of approved cancer therapies, suggesting that many of our other candidates may also represent useful therapeutic targets. For example, our algorithm identified *ERBB2* for *ERBB2*-positive breast cancer [54], *VEGF* for renal cell carcinoma [55], and *MS4A1 (CD20)* for non-Hodgkin's lymphoma [56]. A cDNA element representing *EGFR* was the top gene selected for glioblastoma, where this gene has been shown to be the most frequently amplified protooncogene [57]. For brain, breast, and lung tumors, we subdivided the samples into the previously known histologic and molecular subtypes of these tumors [37,42]. Overlapping but distinct groups of genes were selected for these subtypes (see http://microarray-pubs.stanford.edu/mbp2). cDNA elements representing *ERBB2* made up three of the top five elements selected for the *ERBB2* breast cancer subtype, but none of these were included in the lists for basal and luminal breast cancers. Conversely, CDH3, one of the known basal breast cancer markers, was only identified as a strong marker for basal breast tumors. These findings suggest that overlapping but nonidentical cocktails of molecularly targeted drugs may allow the best distinction between tumor and normal tissues for various tumor subtypes. Among genes not previously well known as tumor markers were several genes with expression patterns highly specific for more than one tumor compared to all normal tissues. These included the receptor tyrosine kinase AXL, which was overexpressed in luminal breast, testicular, and ovarian cancers. Genes such as AXL may represent particularly fruitful candidates for biologically based therapies, as they could potentially have activity against a number of different tumors.

### Identification of Markers of Organ-Specific Tissue Injury

**Figure 6:**
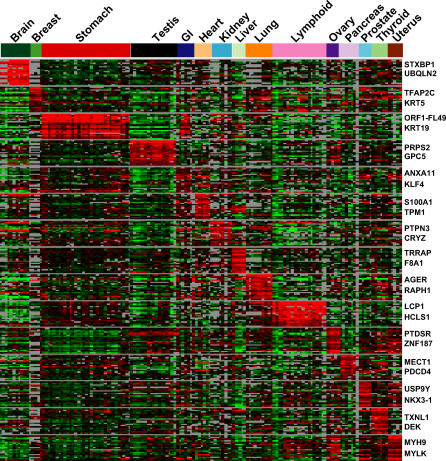
Identification of Markers of Organ-Specific Injury The top-20 CN array elements for each normal tissue were selected using a Student's *t*-test comparing each normal tissue to all other normal tissues. All normal tissues represented by at least five microarray experiments in Figure 4 were included (150 microarrays). Only array elements that passed data quality filters for at least 70% of all normal tissue experiments were considered. Data are displayed as in Figure 4, and the positions of several genes are indicated.

Cytoplasmic and nuclear proteins that are expressed in a tissue-specific fashion are potentially useful markers of specific organ injuries that lead to their release into extracellular spaces. Many such markers are currently used in clinical medicine, including cardiac proteins such as troponin and creatine kinase and “liver enzymes” such as AST and ALT. We therefore assembled a list of approximately 8,500 putative CN genes (UniGene clusters), represented on our DNA microarrays by 15,311 cDNA clones, using an approach analogous to that used to assemble the aggregate list of putative MS proteins in the previous section (see Dataset S3). Focusing on data from 150 microarray analyses of tissue samples representing 15 different normal tissues, we used Student's *t*-test to identify the 20 genes most consistently expressed at a higher level in each of the tissues compared to all others (Figure 6). We found corroborating evidence for the relative tissue specificity of many of the genes that we identified in the literature and in expressed sequence tag abundance data available in SOURCE. For example, two of the genes identified as being relatively highly expressed in breast tissue were *TFAP2C* and *KRT5. TFAP2C,* also known as AP-2γ, plays a role in *ERBB2* expression and has recently been shown to be expressed specifically in myoepithelial cells of the basal ductal and lobular breast epithelia [58], a pattern similar to that of the better-characterized basal ductal marker *KRT5* [59]. Antibodies to a combination of these CN tissue-specific markers may provide useful assays for detection and diagnosis of such varying conditions as traumatic injury, fever of unknown origin, early-stage acute lung injury, and surveillance of metastasis in cancer patients.

## Discussion

We have used DNA microarrays coupled with subcellular fractionation of mRNAs to categorize thousands of genes as either MS or CN. Our classifications agreed well with in silico methods for predicting subcellular localization. The fact that approximately 40% of all previously identified MS proteins, and a similar fraction of MS proteins identified in this work, do not contain predicted SPs or TM domains underscores the continuing importance of experimental approaches to determining the subcellular localization of proteins. It is important to note that both the computational methods and our microarray approach provide only indirect evidence for a specific localization, ultimately requiring direct validation to conclusively determine a protein's localization. Annotation of genes based on both experimental data and in silico predictions will provide the surest assignments. Based on our data, we estimate that approximately 30% of human genes encode MS proteins, a proportion at the upper end of previous estimates [30–32]. This estimate is important, since it increases the possible number of surface-exposed or secreted polypeptides that could serve as useful therapeutic or diagnostic targets, and it highlights the importance of cell–cell communication and extracellular structures in human biology.

We identified a subset of genes encoding known MS or CN proteins whose mRNAs fractionated in an unexpected manner. While one common cause of this phenomenon was incorrect or incomplete annotation of the localization of the proteins encoded by these genes, we also identified intriguing examples that reflected important aspects of the molecular regulation and function of this class of genes. The presence of genes encoding cytoplasmic proteins that function near membrane structures in the group of CN-encoding transcripts with unexpected membrane localization suggests that cells actively sort mRNAs in order to promote their translation at sites where their encoded protein products are required. A recent study in yeast showed that some RNA-binding proteins specifically associate with mRNAs encoding membrane-associated proteins [25], and a similar mechanism may be contributing to the unexpected segregation of some mRNAs that we observed.

The microarray-based approach we used to study subcellular localizations of mRNAs can still be further developed and refined. Simply fractionating more cell lines and extending the approach to whole tissues should allow the classification of many more genes. Technically, it may be possible to refine the specificity of the method by analyzing only those transcripts in the MS and CN fractions that are also associated with polyribosomes. Similarly, approaches identifying mRNAs associated with subcellular organelles, cytoskeletal elements, motor proteins, and RNA-binding proteins will further our understanding of subcellular localization of RNAs and uncover interesting aspects of the biology of the proteins that they encode [25,60–63]. Microarray-based approaches can be particularly useful for organisms that do not have sequenced genomes but for which genomic or expression libraries exist, as in silico-based approaches are not possible in this setting.

Using publicly available gene expression data, we identified hundreds of MS genes with tumor-specific expression patterns. The proteins encoded by these genes represent promising targets for antibody- or small molecule-based therapeutics, for blood based assays for early detection of cancer, for monitoring of treatment responses, and for detecting recurrence following treatment. While there was prior evidence for tumor-specific expression of some of the markers, we identified many novel tumor markers that expand the catalog of potentially useful drug targets. Our approach also allows rational prioritization of known and novel markers for drug development based on relative specificity of expression and absolute transcript abundance, increasing the likelihood of developing successful therapies. Our strategy could be refined by developing methods to focus separately on surface and secreted proteins, as these may be better candidates for therapeutic and diagnostic markers, respectively. Also, expansion of the number of tumor and normal samples with large-scale gene expression data would allow even better accuracy in selecting tumor-specific markers. Our approach might also be extended to anticipate and avoid molecular targets that would risk potential clinical side effects, such as excluding genes highly expressed by hematologic cells to limit bone marrow toxicity or by excluding genes expressed by cardiac myocytes in order to minimize cardiovascular toxicity. The combination of functional annotations such as subcellular localization with systematic data on gene expression in the gamut of normal cells and tissues as well as cancers provides a basis for improved approaches to drug design and diagnosis.

## Materials and Methods

### Subcellular fractionation and RNA isolation.

The methods closely followed those of a previous study [10]. Briefly, we used equilibrium density gradient centrifugation to separate free mRNA and mRNA associated with the rough ER or other membrane structures from a variety of human cell lines [12,64]. Total RNA was isolated from the membrane and cytoplasmic fractions using TRIzol (Life Technologies, Carlsbad, California, United States). For a subset of cell lines, the resulting products were amplified using a linear, in vitro transcription-based, antisense RNA amplification [14].

### Microarray manufacture and hybridization.

DNA microarrays were produced and hybridized as previously described [65] (http://cmgm.stanford.edu/pbrown). To quantitate the distribution of mRNAs between the membrane and cytoplasmic fractions, Cy5-labeled cDNA was prepared from RNA extracted from the rough ER fractions, and Cy3-labeled cDNA was prepared from RNA extracted from the cytoplasmic complement. The cDNA microarrays were produced by the Stanford Functional Genomics Facility and contained a set of approximately 42,000 sequence-confirmed cDNA clones, representing both characterized and uncharacterized genes. Raw images and data from the experiments described here are available at http://microarray-pubs.stanford.edu/mbp2 and the Stanford Microarray Database [66].

### Identification of empirically determined membrane-associated proteins.

Information on experimentally determined subcellular localization of protein products was collected for as many genes as possible. Sources included literature searches and queries of SOURCE [67] (http://source.stanford.edu), which includes subcellular localization information from Swiss-Prot and LocusLink Gene Ontology annotations [15,16,68]. Proteins documented to be secreted, or localized to the ER, golgi, vesicles, or plasma membrane were grouped together as MS, while genes coding for cytosolic or nuclear proteins were designated as CN.

### Bioinformatic analyses.

Analyses were performed as described in the text and figure legends. Perl scripts were used where necessary to facilitate the analyses. For in silico predictions of localization, we used the SignalP program (HMM/Smean score method) [33] for SPs and the TMHMM program (first60 score cutoff greater than 10) [34] for TM domains. More detailed methods, the raw microarray data, and a list containing our experimental gene-product localizations, previously identified protein localization data, and in silico predictions for all genes examined can be found at our Web site (http://microarray-pubs.stanford.edu/mbp2).

## Supporting Information

Dataset S1 Clone Localization Data Experimental, in silico, and known localization data for each well-measured clone.
(7.2 MB XLS)

Dataset S2 MS Clones List of 12,030 MS clones used for the analyses in Figures 4 and 5.
(156 KB TXT)

Dataset S3 CN Clones List of 15,311 CN clones used for the analyses in Figure 6.
(196 KB TXT)

Figure S1 Relationship of Tumors and Normal Tissues Based on the Expression of MS Protein Encoding Genes In order to examine the relationship between tumor and normal specimens stemming from the same tissue, mean centroids were calculated for each tumor and normal tissue group. These were then hierarchically clustered using average linkage clustering. Tumor and normal classes from the same tissue generally clustered together on terminal branches, with a few exceptions (germ cell, pancreatic, ovarian).
(868 KB EPS)

Protocol S1 Supplemental Methods (60 KB DOC)

Table S1 List of Cell Lines Used in This Study The table lists the tissue of origin for each line and its ATTC catalog number (where available).
(30 KB DOC)

Table S2 Gene Ontology (GO) Category Enrichment among MS Genes with Unexpected Subcellular Localization Overrepresentation of GO annotations among characterized MS genes, whose transcripts were enriched in the cytosolic fraction compared to all characterized MS genes, is shown. Corrected *p*-values were calculated using GO-TermFinder.
(48 KB DOC)

### Accession Numbers

The Entrez Gene (http://www.ncbi.nlm.nih.gov/entrez/query.fcgi?db=gene) accession numbers for the genes and proteins discussed in this paper are AFP (174), AXL (558), CDH3 (1001), *CEACAM5* (1048), cyclin B1 (891), cyclin B3 (85417), cyclin E2 (9134), *EGFR* (1956), *ERBB2* (2064), ESR1 (2099), GNAS (2778), GOSR2 (9570), *KLK3* (354), *KRT5* (3852), *MS4A1* (2206), PGR (5241), PTCH (5727), RBP4 (5950), RBP5 (83758), SCYE1 (9255), secretagogin (10590), SEC61B (10952), stomatin (2040), STX17 (55014), *TFAP2C* (7022), and *VEGF* (7422).

## Abbreviations

CN: cytosolic/nuclear
ER: endoplasmic reticulum
MS: membrane-associated/secreted
RBP: retinol-binding protein
SP: signal peptide
TM: transmembrane

## References

[pgen-0020011-b001] Brekke OH, Sandlie I (2003). Therapeutic antibodies for human diseases at the dawn of the twenty-first century. Nat Rev Drug Discov.

[pgen-0020011-b002] Sturgeon C (2002). Practice guidelines for tumor marker use in the clinic. Clin Chem.

[pgen-0020011-b003] Fischbach FT (2003). A manual of laboratory and diagnostic test.

[pgen-0020011-b004] Tashiro K, Tada H, Heilker R, Shirozu M, Nakano T (1993). Signal sequence trap: A cloning strategy for secreted proteins and type I membrane proteins. Science.

[pgen-0020011-b005] Chen H, Leder P (1999). A new signal sequence trap using alkaline phosphatase as a reporter. Nucleic Acids Res.

[pgen-0020011-b006] Peterfy M, Gyuris T, Takacs L (2000). Signal-exon trap: A novel method for the identification of signal sequences from genomic DNA. Nucleic Acids Res.

[pgen-0020011-b007] Kopczynski CC, Noordermeer JN, Serano TL, Chen WY, Pendleton JD (1998). A high throughput screen to identify secreted and transmembrane proteins involved in *Drosophila* embryogenesis. Proc Natl Acad Sci U S A.

[pgen-0020011-b008] Menne KM, Hermjakob H, Apweiler R (2000). A comparison of signal sequence prediction methods using a test set of signal peptides. Bioinformatics.

[pgen-0020011-b009] Moller S, Croning MD, Apweiler R (2001). Evaluation of methods for the prediction of membrane spanning regions. Bioinformatics.

[pgen-0020011-b010] Diehn M, Eisen MB, Botstein D, Brown PO (2000). Large-scale identification of secreted and membrane-associated gene products using DNA microarrays. Nat Genet.

[pgen-0020011-b011] Nickel W (2003). The mystery of nonclassical protein secretion: A current view on cargo proteins and potential export routes. Eur J Biochem.

[pgen-0020011-b012] Mechler BM (1987). Isolation of messenger RNA from membrane-bound polysomes. Methods Enzymol.

[pgen-0020011-b013] Ross DT, Scherf U, Eisen MB, Perou CM, Rees C (2000). Systematic variation in gene expression patterns in human cancer cell lines. Nat Genet.

[pgen-0020011-b014] Wang E, Miller LD, Ohnmacht GA, Liu ET, Marincola FM (2000). High-fidelity mRNA amplification for gene profiling. Nat Biotechnol.

[pgen-0020011-b015] Gasteiger E, Jung E, Bairoch A (2001). SWISS-PROT: Connecting biomolecular knowledge via a protein database. Curr Issues Mol Biol.

[pgen-0020011-b016] Wheeler DL, Church DM, Lash AE, Leipe DD, Madden TL (2002). Database resources of the National Center for Biotechnology Information: 2002 update. Nucleic Acids Res.

[pgen-0020011-b017] Boyle EI, Weng S, Gollub J, Jin H, Botstein D (2004). GO::TermFinder—Open source software for accessing Gene Ontology information and finding significantly enriched Gene Ontology terms associated with a list of genes. Bioinformatics.

[pgen-0020011-b018] Gallagher PG, Romana M, Lieman JH, Ward DC (1995). cDNA structure, tissue-specific expression, and chromosomal localization of the murine band 7.2b gene. Blood.

[pgen-0020011-b019] Wagner L, Oliyarnyk O, Gartner W, Nowotny P, Groeger M (2000). Cloning and expression of secretagogin, a novel neuroendocrine- and pancreatic islet of Langerhans-specific Ca2+-binding protein. J Biol Chem.

[pgen-0020011-b020] Li L, Haynes MP, Bender JR (2003). Plasma membrane localization and function of the estrogen receptor alpha variant (ER46) in human endothelial cells. Proc Natl Acad Sci U S A.

[pgen-0020011-b021] Teo M, Tan L, Lim L, Manser E (2001). The tyrosine kinase ACK1 associates with clathrin-coated vesicles through a binding motif shared by arrestin and other adaptors. J Biol Chem.

[pgen-0020011-b022] Chamorro M, Czar MJ, Debnath J, Cheng G, Lenardo MJ (2001). Requirements for activation and RAFT localization of the T-lymphocyte kinase Rlk/Txk. BMC Immunol.

[pgen-0020011-b023] Barnes EA, Kong M, Ollendorff V, Donoghue DJ (2001). Patched1 interacts with cyclin B1 to regulate cell cycle progression. EMBO J.

[pgen-0020011-b024] Gaulin JF, Fiset A, Fortier S, Faure RL (2000). Characterization of Cdk2-cyclin E complexes in plasma membrane and endosomes of liver parenchyma: Insulin-dependent regulation. J Biol Chem.

[pgen-0020011-b025] Gerber AP, Herschlag D, Brown PO (2004). Extensive association of functionally and cytotopically related mRNAs with Puf family RNA-binding proteins in yeast. PLoS Biol.

[pgen-0020011-b026] Razandi M, Pedram A, Greene GL, Levin ER (1999). Cell membrane and nuclear estrogen receptors (ERs) originate from a single transcript: Studies of ERalpha and ERbeta expressed in Chinese hamster ovary cells. Mol Endocrinol.

[pgen-0020011-b027] Purdue PE, Lazarow PB (2001). Peroxisome biogenesis. Annu Rev Cell Dev Biol.

[pgen-0020011-b028] Kalies KU, Hartmann E (1998). Protein translocation into the endoplasmic reticulum (ER)—Two similar routes with different modes. Eur J Biochem.

[pgen-0020011-b029] Ko YG, Park H, Kim T, Lee JW, Park SG (2001). A cofactor of tRNA synthetase, p43, is secreted to up-regulate proinflammatory genes. J Biol Chem.

[pgen-0020011-b030] Lander ES, Linton LM, Birren B, Nusbaum C, Zody MC (2001). Initial sequencing and analysis of the human genome. Nature.

[pgen-0020011-b031] Stevens TJ, Arkin IT (2000). Do more complex organisms have a greater proportion of membrane proteins in their genomes?. Proteins.

[pgen-0020011-b032] Liu J, Rost B (2001). Comparing function and structure between entire proteomes. Protein Sci.

[pgen-0020011-b033] Nielsen H, Krogh A (1998). Prediction of signal peptides and signal anchors by a hidden Markov model. Proc Int Conf Intell Syst Mol Biol.

[pgen-0020011-b034] Krogh A, Larsson B, von Heijne G, Sonnhammer EL (2001). Predicting transmembrane protein topology with a hidden Markov model: Application to complete genomes. J Mol Biol.

[pgen-0020011-b035] Perou CM, Sorlie T, Eisen MB, van de Rijn M, Jeffrey SS (2000). Molecular portraits of human breast tumours. Nature.

[pgen-0020011-b036] Sorlie T, Perou CM, Tibshirani R, Aas T, Geisler S (2001). Gene expression patterns of breast carcinomas distinguish tumor subclasses with clinical implications. Proc Natl Acad Sci U S A.

[pgen-0020011-b037] Garber ME, Troyanskaya OG, Schluens K, Petersen S, Thaesler Z (2001). Diversity of gene expression in adenocarcinoma of the lung. Proc Natl Acad Sci U S A.

[pgen-0020011-b038] Nielsen TO, West RB, Linn SC, Alter O, Knowling MA (2002). Molecular characterisation of soft tissue tumours: A gene expression study. Lancet.

[pgen-0020011-b039] Bohen SP, Troyanskaya OG, Alter O, Warnke R, Botstein D (2003). Variation in gene expression patterns in follicular lymphoma and the response to rituximab. Proc Natl Acad Sci U S A.

[pgen-0020011-b040] Higgins JP, Shinghal R, Gill H, Reese JH, Terris M (2003). Gene expression patterns in renal cell carcinoma assessed by complementary DNA microarray. Am J Pathol.

[pgen-0020011-b041] Iacobuzio-Donahue CA, Maitra A, Olsen M, Lowe AW, van Heek NT (2003). Exploration of global gene expression patterns in pancreatic adenocarcinoma using cDNA microarrays. Am J Pathol.

[pgen-0020011-b042] Sorlie T, Tibshirani R, Parker J, Hastie T, Marron JS (2003). Repeated observation of breast tumor subtypes in independent gene expression data sets. Proc Natl Acad Sci U S A.

[pgen-0020011-b043] Chen X, Leung SY, Yuen ST, Chu KM, Ji J (2003). Variation in gene expression patterns in human gastric cancers. Mol Biol Cell.

[pgen-0020011-b044] Schaner ME, Ross DT, Ciaravino G, Sorlie T, Troyanskaya O (2003). Gene expression patterns in ovarian carcinomas. Mol Biol Cell.

[pgen-0020011-b045] Sperger JM, Chen X, Draper JS, Antosiewicz JE, Chon CH (2003). Gene expression patterns in human embryonic stem cells and human pluripotent germ cell tumors. Proc Natl Acad Sci U S A.

[pgen-0020011-b046] Linn SC, West RB, Pollack JR, Zhu S, Hernandez-Boussard T (2003). Gene expression patterns and gene copy number changes in dermatofibrosarcoma protuberans. Am J Pathol.

[pgen-0020011-b047] Nielsen TO, Hsu FD, Jensen K, Cheang M, Karaca G (2004). Immunohistochemical and clinical characterization of the basal-like subtype of invasive breast carcinoma. Clin Cancer Res.

[pgen-0020011-b048] Shyamsundar R, Kim YH, Higgins JP, Montgomery K, Jorden M (2005). A DNA microarray survey of gene expression in normal human tissues. Genome Biol.

[pgen-0020011-b049] Liang Y, Diehn M, Watson N, Bollen AW, Aldape KD (2005). Gene expression profiling reveals molecularly and clinically distinct subtypes of glioblastoma multiforme. Proc Natl Acad Sci U S A.

[pgen-0020011-b050] Eisen MB, Spellman PT, Brown PO, Botstein D (1998). Cluster analysis and display of genome-wide expression patterns. Proc Natl Acad Sci U S A.

[pgen-0020011-b051] Seregni E, Coli A, Mazzucca N (2004). Circulating tumour markers in breast cancer. Eur J Nucl Med Mol Imaging.

[pgen-0020011-b052] Brown RW, Campagna LB, Dunn JK, Cagle PT (1997). Immunohistochemical identification of tumor markers in metastatic adenocarcinoma: A diagnostic adjunct in the determination of primary site. Am J Clin Pathol.

[pgen-0020011-b053] Oyama T, Osaki T, Baba T, Nagata Y, Mizukami M (2005). Molecular genetic tumor markers in non-small cell lung cancer. Anticancer Res.

[pgen-0020011-b054] Yarden Y, Baselga J, Miles D (2004). Molecular approach to breast cancer treatment. Semin Oncol.

[pgen-0020011-b055] Yang JC, Haworth L, Sherry RM, Hwu P, Schwartzentruber DJ (2003). A randomized trial of bevacizumab, an anti-vascular endothelial growth factor antibody, for metastatic renal cancer. N Engl J Med.

[pgen-0020011-b056] Hennessy BT, Hanrahan EO, Daly PA (2004). Non-Hodgkin lymphoma: An update. Lancet Oncol.

[pgen-0020011-b057] Hoi Sang U, Espiritu OD, Kelley PY, Klauber MR, Hatton JD (1995). The role of the epidermal growth factor receptor in human gliomas: I. The control of cell growth. J Neurosurg.

[pgen-0020011-b058] Friedrichs N, Jager R, Paggen E, Rudlowski C, Merkelbach-Bruse S (2005). Distinct spatial expression patterns of AP-2alpha and AP-2gamma in non-neoplastic human breast and breast cancer. Mod Pathol.

[pgen-0020011-b059] Bocker W, Bier B, Freytag G, Brommelkamp B, Jarasch ED (1992). An immunohistochemical study of the breast using antibodies to basal and luminal keratins, alpha-smooth muscle actin, vimentin, collagen IV and laminin. Part I: Normal breast and benign proliferative lesions. Virchows Arch A Pathol Anat Histopathol.

[pgen-0020011-b060] Takizawa PA, DeRisi JL, Wilhelm JE, Vale RD (2000). Plasma membrane compartmentalization in yeast by messenger RNA transport and a septin diffusion barrier. Science.

[pgen-0020011-b061] Brown V, Jin P, Ceman S, Darnell JC, O'Donnell WT (2001). Microarray identification of FMRP-associated brain mRNAs and altered mRNA translational profiles in fragile X syndrome. Cell.

[pgen-0020011-b062] Hieronymus H, Silver PA (2003). Genome-wide analysis of RNA-protein interactions illustrates specificity of the mRNA export machinery. Nat Genet.

[pgen-0020011-b063] Shepard KA, Gerber AP, Jambhekar A, Takizawa PA, Brown PO (2003). Widespread cytoplasmic mRNA transport in yeast: Identification of 22 bud-localized transcripts using DNA microarray analysis. Proc Natl Acad Sci U S A.

[pgen-0020011-b064] Diehn M, Bowtell D, Sambrook J (2003). Isolation of membrane-bound polysomal RNA. DNA Microarrays: A molecular cloning manual.

[pgen-0020011-b065] Eisen MB, Brown PO (1999). DNA arrays for analysis of gene expression. Methods Enzymol.

[pgen-0020011-b066] Gollub J, Ball CA, Binkley G, Demeter J, Finkelstein DB (2003). The Stanford Microarray Database: Data access and quality assessment tools. Nucleic Acids Res.

[pgen-0020011-b067] Diehn M, Sherlock G, Binkley G, Jin H, Matese JC (2003). SOURCE: A unified genomic resource of functional annotations, ontologies, and gene expression data. Nucleic Acids Res.

[pgen-0020011-b068] Ashburner M, Ball CA, Blake JA, Botstein D, Butler H (2000). Gene ontology: Tool for the unification of biology. Nat Genet.

