# Supplementary material for: Genome-Scale Identification of Membrane-Associated Human mRNAs
Source: PLoS Genet. 2006 Jan 13;2(1):e11. doi: 10.1371/journal.pgen.0020011 (PMC1326219; doi:10.1371/journal.pgen.0020011)
Supplement: Supplementary file 1 [file pgen.0020011.sd004.doc]

# SUPPLEMENTAL Methods

**Subcellular fractionation and RNA isolation**. The methods closely followed those of a previous study [1]. We used equilibrium density gradient centrifugation to separate free mRNA and mRNA associated with the rough endoplasmic reticulum (rER) or other membrane structures from a variety of human cell lines (see Table S1). Briefly, 5x10^8^ cells were cultured in roller flasks and treated with 50 μM cycloheximide (Sigma) for 10 minutes at 37ºC. Cells were lysed hypotonically using a ball-bearing homogenizer, and fractionated by sedimentation equilibrium as described [2,3]. Degree of separation of membrane-associated and cytosolic ribosomes was monitored using OD260 profiles. Total RNA was isolated from the membrane and cytoplasmic fractions using Trizol (Life Technologies, Inc.) For a subset of cell lines, the resulting products were then amplified using a linear, *in vitro* transcription-based, antisense RNA amplification [4] in order to generate sufficient material for microarray hybridization.

**Microarray manufacture and hybridizations**. DNA microarrays were produced by the Stanford Functional Genomics Facility and hybridized as previously described [5]. To quantitate the distribution of mRNAs between the membrane and cytoplasmic fractions, Cy5-labeled cDNA was prepared from RNA extracted from the rER fractions and Cy3-labeled cDNA was prepared from RNA extracted from the cytoplasmic complement. We used a standard direct dye incorporation labeling protocol (http://cmgm.stanford.edu/pbrown). For most of the arrays, cDNA was synthesized from total RNA in the presence of oligo-dT and fluorescently labeled dUTP (Cy5 or Cy3). For amplified samples, aRNA was converted to fluorescent cDNA by reverse transcription in the presence of a random hexamer oligonucleotide and fluorescent dUTP. Equal amounts of Cy5- and Cy3-labeled cDNA were pooled and hybridized to the microarrays. The cDNA microarrays contained a set of approximately 42,000 sequence-confirmed cDNA clones, representing both characterized and uncharacterized genes, and were scanned at 10 μm resolution using a 4000B GenePix scanner (Axon Instruments Inc.). The resulting images were processed using the GenePix software (Axon Instruments Inc.) and the data were normalized and indexed in the Stanford Microarray Database (SMD). Raw images and data from the experiments described here are publicly available at SMD.

**Identification of empirically determined membrane-associated proteins**. Information on experimentally-determined subcellular localization of protein products was collected for as many genes as possible. The sources for this information included literature searches and queries of SOURCE [6] (http://source.stanford.edu) which includes subcellular localization information from SWISS-PROT and LocusLink GeneOntology annotations [7-9]. Proteins documented to be secreted, or to be localized to the ER, golgi, vesicles, or plasma membrane were grouped together as "membrane-associated/secreted" (MS) while genes coding for cytosolic or nuclear proteins were designated as "cytosolic/nuclear" (CN).

**Bioinformatic analyses.** Stand-alone Perl scripts were used where necessary to facilitate the following analyses:

For the analyses shown in Figure 1A, only genes of known subcellular localization were considered. To calculate a moving average of known membrane-associated proteins using a window size of 151, the fraction of membrane-associated proteins for 151 adjacent genes in Cy5/Cy3 ratio space was computed and plotted as a function of the central gene in the window. The 151 gene window was then moved by one gene on the Cy5/Cy3 axis and the fraction was re-calculated. This process was reiterated until the end of the Cy5/Cy3 distribution was reached.

For the discovery rate analysis depicted in Figure 1B, a representative array was first chosen for each cell line. The moving average analysis described above was performed for each of these arrays and the total number of unique UniGene clusters that were more than 85 percent enriched in the membrane or cytosolic fraction were cataloged. To generate the graph, we started with the first fractionation and plotted the total number of unique UniGene clusters that were represented by the clones more than 85 percent enriched in either fraction. For subsequent fractionations (in random order), we only added the number of unique UniGene clusters that had not been identified in the previously considered fractionations.

In order to identify the largest possible number of MS and CN genes while still retaining good specificity, we began by considering cDNA clones whose Intensity/Background ratio was greater than 2.5 in either channel on at least 3 arrays. We calculated various descriptive statistics (median, mean, minimum, maximum, 25^th^ percentile, 75^th^ percentile) for a number of parameters for every clone across all arrays, including:

- the local percentage of characterized MS genes based on the moving average analysis (see above)
- the base 2 logarithm of the Cy5/Cy3 ratio
- the ratio of intensity to local background for Cy3
- the ratio of intensity to local background for Cy5
- the background-subtracted intensity for Cy3
- the background-subtracted intensity for Cy5

As a final parameter, we included the ratio of the sum of Cy5 background-corrected intensities to the sum of Cy3 background-corrected intensities across all arrays. To identify the best classification approach, receiver-operator curves were generated using each of these parameters. Clones were ranked in descending order by each parameter and a moving average approach was used to identify the local percentage of characterized MS/CN proteins at each point of these distributions. By varying the cut-off percentage of MS/CN encoding genes, we generated clone sets containing varying fractions of genes encoding known MS or CN proteins for which we could calculate a sensitivity and specificity based on the characterized MS and CN genes present on our arrays. Three of the parameters (average log_2_ Cy5/Cy3 ratio, the mean local percentage of characterized MS genes, and the ratio of the sum of Cy5 background-corrected intensities to the Cy3 background-corrected intensities) yielded similarly strong relationships between sensitivity and specificity and we chose the average log_2_ Cy5/Cy3 ratio for the subsequent analyses. Since a subset of the UniGene clusters included on the arrays was represented by two or more elements, we removed all clusters with ambiguous localizations (i.e., clusters that contained clones classified as both MS and CN.) Two enrichment cut-offs were used in subsequent analyses as indicated in the text. The more stringent of these was selected with a local percentage of characterized MS/CN protein cutoff of 82%, while the less stringent was selected with a cutoff of 74%. The results for the less stringent dataset are summarized in Table 1.

For the comparisons between our classifications and *in silico* prediction of localization we first focused on the clones on our microarrays that represented genes with curated, NP protein accessions in LocusLink. We were able to retrieve NP accessions for 5,504 of the well-measured UniGene clusters. The prediction algorithms used were SignalP (HMM/Smean score method) [10] for signal peptides and TMHMM (First60 score cutoff greater than 10) [11] for transmembrane domains. In order to calculate the fraction of proteins within a category that contained a given motif, the overlap between that category and the genes with protein sequences was used. For the Venn diagram analysis, we used a more liberal, non-curated set of representative protein accessions from UniGene. We were able to identify these for 10,006 of the well-measured cDNA clones and extracted them from UniGene via SOURCE. The circles representing our empirical annotations in the Venn diagrams in Figure 3B contain all annotated clones, including those for which protein sequences were not available.

For the Gene Ontology analyses described in the manuscript we used GO-TermFinder [12] to measure the enrichment of Gene Ontology annotations among the various subsets of genes. The background dataset used for calculation of statistical significance was the set of all genes of a given localization (e.g. for the analysis of CN-encoding genes found in the MS fraction, the background dataset was all CN genes that were detectably expressed in any of our fractionation experiments).

For the analysis shown in Figure S1, mean centroids were calculated for each tumor and normal tissue group. These were then hierarchically clustered using average linkage clustering.

**Generation of MS and CN gene lists for tumor and normal tissue marker analyses**. To generate the MS gene list, we began with the list of putative membrane or secreted proteins identified using the less stringent criteria described above. We then removed from this list all of the known genes encoding cytosolic or nuclear proteins that we had curated earlier. We next added any gene encoding a membrane or secreted protein that was identified by our previous database searches but that was not identified as such in our experiments. This aggregate list contained ~7,300 putative MS genes (UniGene clusters), represented on our microarrays by 12,030 cDNA clones. The CN gene list was generated in an analogous fashion. This resulted in a list of ~8,500 putative CN genes (UniGene clusters), represented on our DNA microarrays by 15,311 cDNA clones.

**Tumor and normal tissue MS gene expression analysis.** For the data shown in Figure 4, we first assembled a list of 745 previously published microarray analyses of human tumors and normal tissues (see references in manuscript). We then used our MS gene list to select only those MS elements for which at least 70% of the features across all samples had pixel-based regression ratios greater than 0.6. The logarithm of the ratio of background-subtracted Cy5 fluorescence to background-subtracted Cy3 fluorescence was calculated. Next, the values for each array and each gene were median centered (in that order), and only cDNA array elements for which at least three measurements differed by more than 3-fold from the median were included in the subsequent analysis. For clarity of display, arrays were arranged by the order derived from clustering their mean centroids. Mean centroids for tumor and normal samples within each of eleven groups (brain, breast, stomach, germ cell, kidney, lung, lymphoid, ovary, pancreas, soft tissue, remaining normal tissues) were calculated and hierarchically clustered. Arrays were then individually clustered within each of the eleven groups and these were assembled in the order defined by the mean centroid clustering to create Figure 4.

**Identification of membrane-associated or secreted tumor markers.** For the tumor marker analysis in Figure 5, we included only those MS features on a given array that had pixel-based regression ratios greater than 0.6. We next considered only array elements that passed this data quality filter for at least 40% of normal tissues and at least 50% of one or more of the tumor classes. For each tumor type, array elements were ranked based on the difference between the median expression in tumor samples and the 95th percentile expression level across all normal tissue samples. Breast and lung tumors were further subdivided into their molecularly or histologically recognized subgroups.

**Identification of markers of organ-specific injury.** For this analysis we limited our dataset to the 150 microarray analyses of normal tissue samples from Figure 4 that represented tissues with a minimum of 5 microarrays. We included only those CN array elements that had pixel-based regression ratios greater than 0.6 on at least 70% of these arrays. We then used a Student’s t-test to identify the 20 genes most consistently expressed at a higher level in each of the normal tissues compared to all others.

**References:**

1. Diehn M, Eisen MB, Botstein D, Brown PO (2000) Large-scale identification of secreted and membrane-associated gene products using DNA microarrays. Nat Genet 25: 58-62.

2. Mechler BM (1987) Isolation of messenger RNA from membrane-bound polysomes. Methods Enzymol 152: 241-248.

3. Diehn M (2003) Isolation of membrane-bound polysomal RNA. In: Bowtell D, Sambrook J, editors. DNA Microarrays: a molecular cloning manual. Cold Spring Harbor, NY: Cold Spring Harbor Laboratory Press.

4. Wang E, Miller LD, Ohnmacht GA, Liu ET, Marincola FM (2000) High-fidelity mRNA amplification for gene profiling. Nat Biotechnol 18: 457-459.

5. Eisen MB, Brown PO (1999) DNA arrays for analysis of gene expression. Methods Enzymol 303: 179-205.

6. Diehn M, Sherlock G, Binkley G, Jin H, Matese JC, et al. (2003) SOURCE: a unified genomic resource of functional annotations, ontologies, and gene expression data. Nucleic Acids Res 31: 219-223.

7. Gasteiger E, Jung E, Bairoch A (2001) SWISS-PROT: connecting biomolecular knowledge via a protein database. Curr Issues Mol Biol 3: 47-55.

8. Wheeler DL, Church DM, Lash AE, Leipe DD, Madden TL, et al. (2002) Database resources of the National Center for Biotechnology Information: 2002 update. Nucleic Acids Res 30: 13-16.

9. Ashburner M, Ball CA, Blake JA, Botstein D, Butler H, et al. (2000) Gene ontology: tool for the unification of biology. The Gene Ontology Consortium. Nat Genet 25: 25-29.

10. Nielsen H, Krogh A (1998) Prediction of signal peptides and signal anchors by a hidden Markov model. Proc Int Conf Intell Syst Mol Biol 6: 122-130.

11. Krogh A, Larsson B, von Heijne G, Sonnhammer EL (2001) Predicting transmembrane protein topology with a hidden Markov model: application to complete genomes. J Mol Biol 305: 567-580.

12. Boyle EI, Weng S, Gollub J, Jin H, Botstein D, et al. (2004) GO::TermFinder--open source software for accessing Gene Ontology information and finding significantly enriched Gene Ontology terms associated with a list of genes. Bioinformatics 20: 3710-3715.
