# Supplementary material for: Genome-Scale Identification of Membrane-Associated Human mRNAs
Source: PLoS Genet. 2006 Jan 13;2(1):e11. doi: 10.1371/journal.pgen.0020011 (PMC1326219; doi:10.1371/journal.pgen.0020011)
Supplement: Supplementary file 2 [file pgen.0020011.st001.doc]

**Table S1:**

| Name | **Tissue of Origin** | **ATCC** |
| --- | --- | --- |
| Colo 205 | Colorectal adenocarcinoma | CCL-222 |
| Hep G2 | Hepatocellular carcinoma | HB-8065 |
| Hs 578T | Breast carcinoma | HTB-126 |
| Jurkat | Acute T cell leukemia | TIB-152 |
| K-562 | Chronic myelogneous leukemia/Erythroleukemia | CCL-243 |
| **LNCaP** | Prostate carcinoma | CRL-1740 |
| **MCF7** | Breast adenocarcinoma | HTB-22 |
| MOLT-4 | Acute T cell leukemia | CRL-1582 |
| OVCAR-3 | Ovarian adenocarcinoma | HTB-161 |
| RPMI 8226 | Myeloma (B cell) | CCL-155 |
| VDSO | Lymphoblastoid | -- |
