# Supplementary material for: Genome-Scale Identification of Membrane-Associated Human mRNAs
Source: PLoS Genet. 2006 Jan 13;2(1):e11. doi: 10.1371/journal.pgen.0020011 (PMC1326219; doi:10.1371/journal.pgen.0020011)
Supplement: Supplementary file 3 [file pgen.0020011.st002.doc]

**Table S2**:

| Ontology | **GO Term** | **Example Genes** | **p value** |
| --- | --- | --- | --- |
| **Process** | intracellular transport | RAB6A, AP3S1, AP3B1, GOSR1, HOMER3 | 4.62E-14 |
|  | intracellular protein transport | COG1, RAB9A, APBA3, AP1M1, AP3M1 | 8.36E-14 |
|  | protein transport | SEC61G, AKAP5, STN2, TOM1L2, AP3S2 | 4.43E-13 |
|  | Golgi vesicle transport | KIF1C, GOSR1, TOM1L1, COG1, GOSR2 | 1.14E-06 |
|  | vesicle-mediated transport | STN2, DNM1L, TOM1L2, GGA1, AP2A2 | 5.68E-06 |
|  | secretory pathway | SNAP23, AP3B2, TOM1L2, GGA1, TRAPPC1 | 9.54E-06 |
|  | intra-Golgi transport | COG1, COG2, COG5, GGA2, GGA3 | 9.50E-05 |
|  | organelle organization and biogenesis | FLNB, RDX, COG1, RHOA, PARD6B | 2.13E-03 |
|  | small GTPase mediated signal transduction | RAB36, RHOA, RAB9A, RIT1, RAP2A | 5.68E-03 |
|  | cytoplasm organization and biogenesis | PLD2, ADD1, CDC42EP2, ECGF1, DOCK2, SGCD | 2.44E-02 |
| **Component** | Golgi apparatus | AP3S1, AP3B1, GOSR1, COG1, GOLGA1 | 1.61E-12 |
|  | intracellular | RRAS2, AP3S1, RDX, GOSR1, COG1 | 2.25E-08 |
|  | cytoplasm | IDS, GBF1, DSC1, GBA, DMD | 4.08E-08 |
|  | membrane coat | AP3S1, AP3S2, AP2A2, SEC24A, AP3M2 | 4.28E-04 |
|  | vesicle coat | AP3M1, AP1GBP1, SEC23B, COPZ1, STN2 | 4.28E-04 |
|  | cell | SLC39A10, RRAS2, AP3S1, RDX, MMD | 4.33E-04 |
|  | clathrin coat | AP1M1, AP3M1, AP1GBP1, COPZ1, STN2 | 2.66E-03 |
|  | clathrin vesicle coat | AP3S1, AP3S2, AP2A2, AP3M2, AP1M1 | 2.66E-03 |
|  | coated vesicle | SEC24A, LOC51760, TRAPPC4, AP1GBP1, SEC23B | 8.13E-03 |
|  | Golgi stack | TOM1L2, TRAPPC4, GNAS, GGA1, GGA2 | 1.67E-02 |
|  | cytoskeleton | FLNB, RDX, KIF1C, RHOA, EVA1 | 2.54E-02 |
|  | cytoplasmic vesicle | AP3S1, VAMP2, SYT1, AP3M2, AP1M1 | 2.54E-02 |
|  | clathrin-coated vesicle | SYT1, AP3M2, AP1M1, TRAPPC4, AP3M1 | 4.46E-02 |
| **Function** | protein transporter activity | RAB6A, AP3S1, AP3B1, GOSR1, COG1 | 2.07E-16 |
|  | GTPase activity | RRAS2, RAB36, RHOA, RAB9A, RAP2A | 3.93E-06 |
|  | GTP binding | RIT1, RAP2A, GNA15, GNAS, MRAS | 6.60E-04 |
|  | guanyl nucleotide binding | RAP1B, ARFRP1, RAB1A, DNM1L, GNA13 | 6.60E-04 |
